# Effects of Smoking on Regional Homogeneity in Mild Cognitive Impairment: A Resting-State Functional MRI Study

**DOI:** 10.3389/fnagi.2020.572732

**Published:** 2020-11-19

**Authors:** Tianyi Zhang, Xiao Luo, Qingze Zeng, Yanv Fu, Zheyu Li, Kaicheng Li, Xiaocao Liu, Peiyu Huang, Yanxing Chen, Minming Zhang, Zhirong Liu, the Alzheimer’s Disease Neuroimaging Initiative (ADNI)

**Affiliations:** ^1^Department of Neurology, The Second Affiliated Hospital of Zhejiang University School of Medicine, Hangzhou, China; ^2^Department of Radiology, The Second Affiliated Hospital of Zhejiang University School of Medicine, Hangzhou, China

**Keywords:** smoking, amnestic mild cognitive impairment, Alzheimer’s disease, resting-state functional magnetic resonance, regional homogeneity

## Abstract

**Background:**

Smoking is a modifiable risk factor for Alzheimer’s disease (AD). However, smoking-related effects on intrinsic brain activity in high-risk AD population are still unclear.

**Objective:**

We aimed to explore differences in smoking effects on brain function between healthy elderly and amnestic mild cognitive impairment (aMCI) patients using ReHo mapping.

**Methods:**

We identified 64 healthy elderly controls and 116 aMCI patients, including 98 non-smoking and 18 smoking aMCI. Each subject underwent structural and resting-state functional MRI scanning and neuropsychological evaluations. Regional homogeneity (ReHo) mapping was used to assess regional brain synchronization. After correction for age, gender, education, and gray matter volume, we explored the difference of ReHo among groups in a voxel-wise way based on analysis of covariance (ANCOVA), followed by *post hoc* two-sample analyses (*p* < 0.05, corrected). Further, we correlated the mean ReHo with neuropsychological scales.

**Results:**

Three groups were well-matched in age, gender, and education. Significant ReHo differences were found among three groups, located in the left supramarginal gyrus (SMG) and left angular gyrus (AG). Specifically, non-smoking aMCI had lower ReHo in SMG and AG than smoking aMCI and controls. By contrast, smoking aMCI had greater AG ReHo than healthy controls (*p* < 0.05). Across groups, correlation analyses showed that left AG ReHo correlated with MMSE (*r* = 0.18, *p* = 0.015), clock drawing test (*r* = 0.20, *p* = 0.007), immediate recall (*r* = 0.36, *p* < 0.001), delayed recall (*r* = 0.34, *p* < 0.001), and auditory verbal learning test (*r* = 0.20, *p* = 0.007).

**Conclusion:**

Smoking might pose compensatory or protective effects on intrinsic brain activity in aMCI patients.

## Introduction

Alzheimer’s disease (AD) is one of the most common neurodegenerative diseases characterized by progressive cognitive impairment. Currently, no specific medicine could reverse the AD course. Accordingly, early diagnosis and reduction of risk factors are clinical strategies to delay disease progression. Amnestic MCI (amnestic mild cognitive impairment, aMCI) is the transitional stage between normal aging and AD. Although 10–15% aMCI patients progress to AD per year, more than half of aMCI patients do not progress even over 10 years of follow-up (Visser et al., 2006; Drago et al., 2011). Thus, it is essential to determine those aMCI patients who are having a higher risk of clinical progression. Based on previous work, many modifiable risk factors contribute to AD, such as smoking. Although smoking cessation may prevent AD progression, the underlying neuronal basis is still unclear.

It is still controversial whether smoking has a positive or negative effect on the brain. For example, studies reported that smoking is negatively related to Parkinson’s disease and might have protective effects on neurons (Wirdefeldt et al., 2011). By contrast, cerebrovascular research showed that smoking is a risk factor for cerebral small vascular disease (Khan et al., 2007). Previous studies indicated that smokers were 1.9–4.3 times more likely to develop AD than non-smokers (Aggarwal et al., 2006; Anstey et al., 2007; Reitz et al., 2007). The Honolulu–Asia Aging Study further pointed out that the smoking amount had a strong dose-response relationship with AD and neuritic plaques (Tyas et al., 2003). Mechanically, animal studies showed that smoking-related oxidative stress might directly promote the amyloid pathway and tau phosphorylation (Durazzo et al., 2014). With the advancement of neuroimaging, accumulating studies have demonstrated the effects of smoking on the brain.

Structurally, smoking is linked to thinner anterior cingulum, prefrontal lobe, and orbitofrontal gyrus, as well as smaller occipital, temporal lobe, and cerebellum gray matter volume (Brody et al., 2004; Gallinat et al., 2006; Kuhn et al., 2012; Fritz et al., 2014; Karama et al., 2015). Functionally, an established method, namely, regional homogeneity (ReHo), has been widely applied to explore regional brain activity, based on the consistency of the brain activity of neighboring voxels (Zang et al., 2004). Notably, based on ReHo mapping, previous studies demonstrated that aMCI and AD had abnormal brain spontaneous activations in the medial prefrontal cortex, posterior cingulate gyrus/precuneus, and inferior parietal lobule (IPL) (Zhang et al., 2012). Also, a smoking-related functional MRI (fMRI) study showed that heavy smokers exhibited decreased ReHo in prefrontal regions and increased ReHo in the insula and posterior cingulate cortex (Yu et al., 2013). Smokers are not a minority of aMCI patients. However, it is still unclear whether smoking is a risk or protective factor for the brain function of aMCI *in vivo*.

To cover these gaps, we aimed to explore smoking effects on brain function in 64 elderly controls and 116 aMCI patients using ReHo mapping. Based on previous work, we hypothesized that aMCI smoking groups might have more severe brain function impairment than aMCI non-smoking groups, especially in regions susceptible to nicotine or AD-related pathologies.

## Materials and Methods

### Alzheimer’s Disease Neuroimaging Initiative

Data used in this study was obtained from the Alzheimer’s disease Neuroimaging Initiative (ADNI) database^1^. The ADNI was launched in 2003 by the National Institute on Aging (NIA), the National Institute of Biomedical Imaging and Bioengineering (NIBIB), the Food and Drug Administration (FDA), private pharmaceutical companies, and non-profit organizations, as a $60 million, 5-year public–private partnership. The primary goal of ADNI has been to test whether serial MRI, positron emission tomography (PET), other biological markers, and clinical and neuropsychological assessment can be combined to measure the progression of aMCI and early AD. Determination of sensitive and specific markers of very early AD progression is intended to aid researchers and clinicians in developing new treatments and monitoring their effectiveness, as well as lessening the time and cost of clinical trials.

### Participants

The Institutional Review Board approved the study of all of the participating institutions. Informed written consent was obtained from all participants at each site. Based on the ADNI GO and ADNI 2 databases, we identified 93 right-handed cognitively intact healthy participants and 187 aMCI patients. We downloaded data from the ADNI database before April 2019. Each subject underwent 3D T1 structural and rsfMRI scanning and neuropsychological evaluation. For the ADNI, to be classified as healthy controls, the subject had a Mini-Mental State Examination (MMSE) between 24 and 30 (inclusive) and a clinical dementia rating (CDR) score of 0. Additionally, there were no signs of depression (Geriatric Depression Scale, GDS < 6) or dementia. To be classified as aMCI, the subject had an MMSE between 24 and 30 (inclusive), a memory complaint, objective memory loss defined as scoring below an education-adjusted cutoff score on delayed recall of the Wechsler Memory Scale (WMS-R) logical memory test, a CDR score of 0.5, general cognition preserved, stable medication, and no signs of depression. We excluded subjects with a history of obvious head trauma, other neurological or significant psychiatric disorders, alcohol/drug abuse, and a significant vascular disease risk history defined as Hachinski Ischemia Scale (HIS) >4. Then, we classified aMCI patients into smoking aMCI and non-smoking aMCI based on self-report smoking history. Smoking is defined by the subjective self-report smoking history from the medical record.

Finally, we exclude eight subjects due to head motion (three healthy controls, four non-smoking aMCI group, and one smoking aMCI), 14 subjects due to clinical depression (two healthy controls, nine non-smoking aMCI group, and three smoking aMCI), 29 subjects due to non-conformity diagnosis between the scales (nine in healthy controls, 19 non-smoking aMCI, and one smoking aMCI smoking), and 49 subjects due to incomplete scale and imaging data (15 in healthy controls, 21 non-smoking aMCI, and 13 smoking aMCI). Finally, 64 healthy controls, 18 smoking aMCI, and 98 non-smoking aMCI entered subsequent analyses. A flowchart of screening processes and detailed smoking information is found in Supplementary Material 1.

### Neuropsychologic Testing

Each subject underwent a neuropsychologic battery to assess general mental status (MMSE and CDR) and other cognitive domains, including memory (Auditory Verbal Learning Test, AVLT; Immediate Story Retell, IST; Delayed Story Retell, DST), processing speed (Trail-Making Test, Part A, TMT-A) (Miner and Ferraro, 1998), visuospatial function (Clock-Drawing Test, CDT), executive function (Trail-Making Test, Part B, TMT-B), and language (Semantic Verbal Fluency, SVF).

### Data Acquisition

Based on the 3T Philips MRI scanner, structural images were acquired using the MPRAGE T1-weighted sequence with the following parameters: repetition time (TR) = 2300 ms; echo time (TE) = 2.98 ms; inversion time (TI) = 900 ms; 170 sagittal slices; within-plane FOV = 256 mm × 240 mm; voxel size = 1.1 mm × 1.1 mm × 1.2 mm; flip angle = 9°; bandwidth = 240 Hz/pix. The rsfMRI images were obtained using an echo-planar imaging sequence with the following parameters: 140 time points; TR = 3000 ms; TE = 30 ms; flip angle = 80°; number of slices = 48; slice thickness = 3.3 mm; spatial resolution = 3.31 mm × 3.31 mm × 3.31 mm; matrix = 64 × 64. According to the human scan protocol of the ADNI database, all subjects should keep their eyes open with fixation (focus on a point on the mirror) during the entire resting-state fMRI scan.

### Imaging Preprocessing

We preprocessed imaging data using the DPABI, implanted in Statistical Parametric Mapping software (SPM8) (Wellcome Trust Center). The first ten image volumes of rsfMRI scans were discarded for the signal equilibrium and subject’s adaptation to the scanning noise. The remaining 130 images were corrected for timing differences between each slice and head motion (6-parameter rigid body). Datasets with more than 2.0 mm maximum displacement in any of the x, y, or z directions or 2.0° of any angular motion were discarded (eight subjects excluded, including three healthy controls, four non-smoking aMCI, and one smoking aMCI). Subsequently, based on rigid-body transformation, we co-registered the T1W image to the mean rsfMRI image, then spatially normalized to the Montreal Neurological Institute (MNI) stereotactic space, and resampled into 3 mm × 3 mm × 3 mm. Finally, linear trends and temporally filter (0.01 Hz < f < 0.08 Hz) were performed. To remove any residual effects of motion and other non-neuronal factors, six head motion parameters, WM signal, and cerebrospinal fluid signal were used as nuisance variables in the functional connectivity analysis. The output of these preprocessing steps was a 4D residual functional volume in native functional space for each subject. These 4D native data were registered to the MNI152 space with a 2-mm resolution based on the affine transformation. Given the disputation of removing the global signal in the preprocessing of rsfMRI data, we omit to regress the global signal out.

### Regional Homogeneity Mapping

The preprocessed functional images were imported into the DPABI toolbox (Yan et al., 2016) to calculate ReHo. According to the hypothesis that intrinsic brain activity appears in a mass or region made up of many cluster volumes, Zang et al. (2004) proposed ReHo. This metric is calculated based on Kendall’s coefficient of concordance (KCC) of the time series of a given voxel with its nearest neighbors (by Kendall, 2010). A higher ReHo for a given voxel indicates a higher regional coherence within a cluster made up of the voxel and its nearest neighbors.

We calculated ReHo using preprocessed 4D rsfMRI data without spatial smoothing. All individual ReHo maps were computed and standardized into ReHo *Z*-value by subtracting the mean voxel-wise ReHo obtained for the entire brain (i.e., global ReHo) and then dividing the resultant value by the standard deviation (Zuo et al., 2013). Finally, generated ReHo maps were spatially smoothed with a 6 mm full width at half maximum (FWHM) Gaussian kernel.

The amplitude of low-frequency fluctuation (ALFF) and fractional ALFF (fALFF) are indexes to detect regional spontaneous brain activity (Zou et al., 2008). Degree centrality (DC) measures the centrality or importance of individual clusters in the brain (Zuo et al., 2012). These indexes describe brain activity from different perspectives. Further, we processed these results based on the DPABI toolbox (Yan et al., 2016), and we extracted the values of ALFF, fALFF, and DC based on the region of interest (ROI).

### Statistical Analysis

We expressed quantitative variables as mean ± standard deviation and categorical variables as absolute and relative frequencies. All statistical analyses were performed using IBM SPSS 23 statistical software on Windows. Group differences in age, education attainment, and neuropsychological scores were examined by one-way analyses of variance (ANOVA). Using Bonferroni correction, a *post hoc* pair-wise *T*-test was performed if ANOVA was significant (*p* < 0.05). We analyzed categorical variables used a Chi -square test. We assessed the normal distribution and variance homogeneity of neuropsychiatric scales (Supplementary Material 2). We have done z-conversion for all the neuropsychologic tests to check the data to conform to the normal distribution.

The statistical analyses of ReHo were performed using Resting-State fMRI Data Analysis Toolkit (REST). Firstly, ANOVA was conducted to identify the ReHo differences among the healthy controls, smoking aMCI, and non-smoking aMCI (multiple-comparison correction based on Gaussian random field, at a significance level of *p* < 0.05 at height and *p* < 0.05 at cluster level). For clusters displaying ReHo differences among groups, we performed *post hoc* 2-sample *t*-tests. We also investigated the associations of imaging measures and behavioral data in the three groups separately and aMCI patients. Notably, correlation analyses were performed only within regions exhibiting significant ReHo differences among the three groups. The statistical significance level of correlation analyses was chosen as *p* < 0.05 (uncorrected) since these analyses were exploratory.

## Results

### Demographics

No difference in age and education among the three groups (*p* > 0.05) existed (Table 1). However, the number of women in the aMCI smoking group is relatively deficient (*p* = 0.01). Thus, we considered gender as a covariate in the following imaging analyses. There is no significant difference in MMSE scores between the three groups (*p* = 0.07). In further neuropsychological tests, three groups have significant differences in memory and language function [IST (*p* < 0.001) and DST (*p* < 0.001), AVLT (*p* < 0.001), and SVF (*p* = 0.003)]. Healthy controls had higher scores of the IST, DST, and AVLT than aMCI groups. No differences in cognitive performance between smoking aMCI and non-smoking aMCI existed (Supplementary Material 3).

**TABLE 1:** Comparison of demographic information and behavioral data among three groups.

	**HC (*n* = 64)**	**Non-smoking aMCI (*n* = 98)**	**Smoking aMCI (*n* = 18)**	***F*/ χ2-value**	***p*-value**
Age (years)	75.75 ± 5.98	73.67 ± 6.78	74.61 ± 8.84	1.86	0.16
Education (years)	16.44 ± 2.50	16.80 ± 2.60	15.72 ± 2.32	1.48	0.23
Sex (F/M)	37/27	39/59	4/14	9.09	0.01*
MMSE	28.98 ± 1.37	28.42 ± 1.59	28.33 ± 1.75	2.75	0.07
**Visuospatial**					
CDT	4.77 ± 0.50	4.50 ± 0.91	4.56 ± 0.51	2.44	0.09
**Memory**					
IST	15.67 ± 3.28	11.24 ± 4.15	11.72 ± 4.28	26.21	<0.001^*ab*^
DST	14.63 ± 3.45	9.25 ± 4.38	10.16 ± 4.31	34.56	<0.001^*ab*^
AVLT	46.72 ± 11.51	37.92 ± 11.86	38.06 ± 10.98	11.70	<0.001^*ab*^
**Language**					
SVF	21.63 ± 4.91	18.95 ± 4.68	20.67 ± 5.77	5.98	0.003^*a*^
**Attention**					
TMT-A	33.22 ± 11.45	35.44 ± 13.45	33.28 ± 10.42	0.70	0.50
**Execution**					
TMT-B	85.41 ± 59.33	95.54 ± 51.54	97.94 ± 62.08	0.75	0.47

Data are presented as mean ± standard deviation, HC, healthy controls; aMCI, amnestic mild cognitive impairment; F, female; M, male; MMSE, Mini-Mental State Examination; CDT, clock drawing test; IST, immediate story retell; DST, delayed story retell; AVLT, auditory verbal learning test; SVF, semantic verbal fluency; TMT-A, Trail-Making Test, Part A; TMT-B, Trail-Making Test, Part B. *Because of the small number of female smokers, we will further use ANCOVA to eliminate the effect of sex. ^*a*^*Post hoc* paired comparisons showed significant group differences between HC and aMCI non-smoker after LSD correction. ^*b*^*Post hoc* paired comparisons showed significant group differences between HC and aMCI smoker, after LSD correction.

### Smoking aMCI Group Had the Highest Intrinsic Brain Activity Among Groups

**FIGURE 1:**
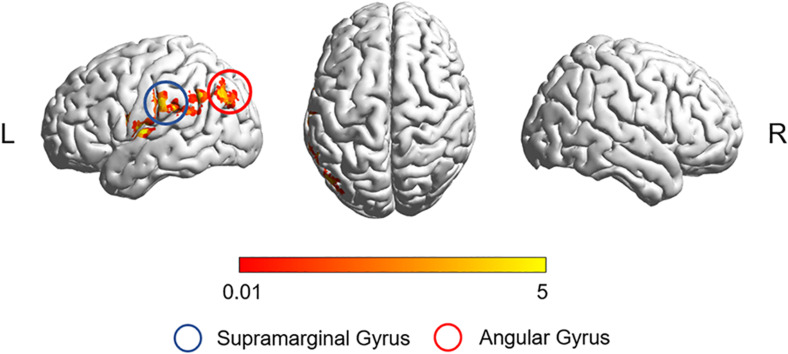
The ReHo difference among the three groups. The figure shows the difference in the ReHo index after adjustment for age, sex, education, and gray matter volume in healthy controls, non-smoking aMCI group, and smoking aMCI group. The three groups have significant differences in the left SMG and left AG (GRF corrected, *p* < 0.05 at height and *p* < 0.05 at cluster level). ReHo, regional homogeneity; aMCI, amnestic mild cognitive impairment; SMG, supramarginal gyrus; AG, angular gyrus.

**FIGURE 2:**
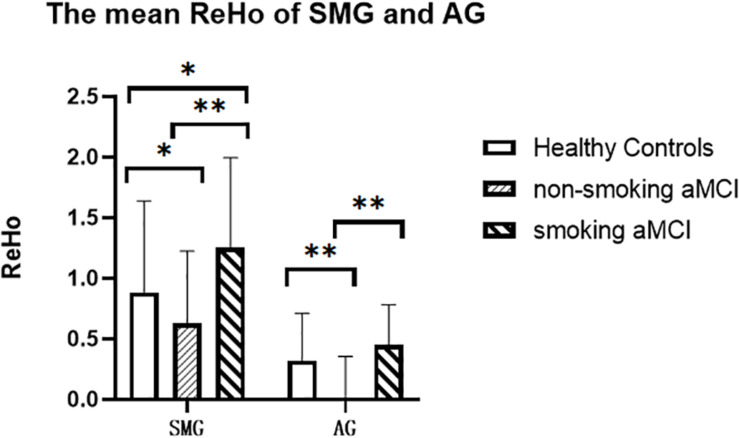
The mean ReHo of the left SMG and left AG of the three groups. The figure shows the mean ReHo in the left SMG and left AG of the healthy controls, aMCI non-smoking group, and aMCI smoking group. ReHo in the aMCI smoking group is significantly higher than those in the aMCI non-smoking group (*p* < 0.001). ReHo in the aMCI non-smoking group is lower than those in the healthy controls (*p* = 0.018). ReHo, regional homogeneity; SMG, supramarginal gyrus; AG, angular gyrus; aMCI, amnestic mild cognitive impairment. *p < 0.05, LSD corrected. **p < 0.01, LSD corrected.

After correction for age, gender, education, and gray matter volume, analysis of covariance (ANCOVA) of ReHo mapping showed that three groups are different in the left supramarginal gyrus (SMG) and left angular gyrus (AG) (*p* < 0.05, GRF corrected) (Figure 1 and Table 2). *Post hoc* results showed that non-smoking aMCI had lower left AG ReHo than smoking aMCI and controls (*p* < 0.001), while there was no difference between the smoking aMCI and controls (*p* = 0.17). Additionally, the non-smoking aMCI group had lower left SMG ReHo than controls (*p* = 0.018). In the aMCI patients, the aMCI smoking group had higher left SMG ReHo (*p* < 0.001). The aMCI smoking group had greater SMG ReHo than the aMCI non-smoking group and controls, while the aMCI non-smoking group had lower SMG ReHo than healthy controls (*p* = 0.04) (Figure 2). Notably, after correction for age, gender, education, history of hypertension, diabetes, drinking and hearing loss, and gray matter volume, ANCOVA of ReHo mapping was mostly unchanged (Supplementary Material 4). We also re-assessed the brain activity differences of the amplitude of low-frequency fluctuation (ALFF), fractional ALFF (fALFF), and degree centrality (DC) among the three groups (Supplementary Material 5).

**TABLE 2:** Brain areas with significant ReHo difference among the three groups.

**Region**	**Cluster voxels**	**MNI coordinate**			**Peak intensity**
		**X**	**Y**	**Z**	
**ANCOVA between three groups**					
Left SMG	128	−63	−21	33	9.72
Left AG	47	−51	−72	36	7.24
**Two sample *t*-test**					
Healthy controls VS aMCI non-smoking group					
Left AG	400	−63	−21	33	−3.80
**aMCI non-smoking group VS aMCI smoking group**					
Left AG	340	−54	−51	27	4.18

*MNI, Montreal Neurological Institute; X Y Z coordinates of the primary peak locations in the MNI space; SMG, supramarginal gyrus; AG, angular gyrus.*

We also did two-way ANCOVA to investigate both the main effects of smoking and aMCI status and further their interactions on cognitive performances and brain ReHo metrics. We found the interaction effect in four brain regions, including the AG, SMG, precentral gyrus, and superior temporal gyrus (Supplementary Material 6).

### Correlations Between ReHo and Neuropsychological Scores

**FIGURE 3:**
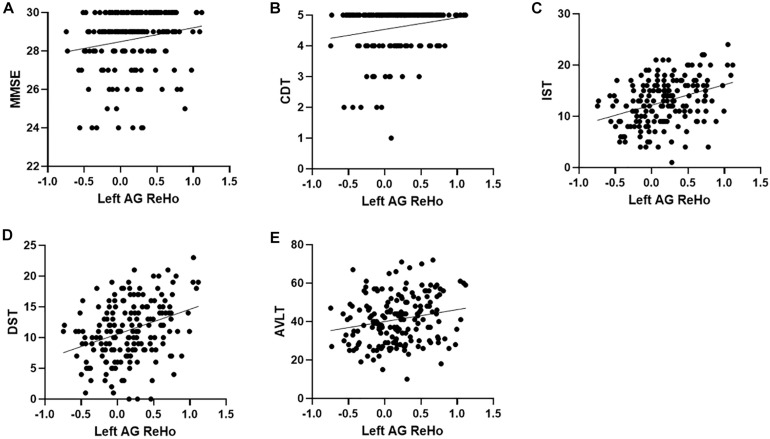
Correlation between ReHo and neuropsychological scores. **(A–E)**, respectively represent the correlation between the ReHo of the left AG and MMSE (*r* = 0.18, *p* = 0.014), CDT (*r* = 0.20, *p* = 0.007), IST (*r* = 0.36, *p* < 0.001), DST (*r* = 0.34, *p* < 0.001), and AVLT (*r* = 0.20, *p* = 0.007), all of which are positively correlated. ReHo, regional homogeneity; MMSE, Mini-Mental State Examination; CDT, clock drawing test; IST, immediate story retell; DST, delayed story retell; AVLT, auditory verbal learning test; SMG, supramarginal gyrus; AG, angular gyrus.

Across all the groups, correlation results showed that left AG ReHo related to MMSE (*r* = 0.18, *p* = 0.014), CDT (*r* = 0.20, *p* = 0.007), IST (*r* = 0.36, *p* < 0.001), DST (*r* = 0.34, *p* < 0.001), and AVLT (*r* = 0.20, *p* = 0.007). The ReHo in the left SMG is also correlated with DST (*r* = 0.16, *p* = 0.04) and IST (*r* = 0.16, *p* = 0.03) (Table 3 and Figure 3). We further compared the correlation between ReHo and scale in each group. Correlation results showed that left AG ReHo is related to IST (*r* = 0.44, *p* < 0.001) and DST (*r* = 0.31, *p* = 0.013) in healthy controls and is related to MMSE (*r* = 0.30, *p* = 0.002) in the non-smoking aMCI (Supplementary Material 7). We also explore the correlation relationships among the smoking year and amount, ReHo value, and clinical scales, but no significant correlations were found (Supplementary Material 8).

**TABLE 3:** Correlations between ReHo and neuropsychological scores.

	**Left SMG**		**Left AG**	
	**Correlation coefficient**	***p*-value**	**Correlation coefficient**	***p-*value**
**MMSE**	−0.02	0.80	0.18	0.014^*b*^
**Visuospatial**				
CDT	−0.03	0.74	0.20	0.007^*b*^
**Memory**				
IST	0.16	0.04	0.36	<0.001^*ab*^
DST	0.16	0.03	0.34	<0.001^*ab*^
AVLT	0.13	0.08	0.20	0.007^*b*^
**Language**				
SVF	0.06	0.43	0.13	0.081
**Attention**				
TMT-A	0.01	0.90	−0.08	0.298
**Execution**				
TMT-B	0.10	0.20	−0.09	0.210

*MMSE, Mini-Mental State Examination; CDT, clock drawing test; IST, immediate story retell; DST, delayed story retell; AVLT, auditory verbal learning test; SVF, semantic verbal fluency; TMT-A, Trail-Making Test, Part A; TMT-B, Trail-Making Test, Part B. The left AG ReHo is related to CDT, immediate, delayed recall, and AVLT. ^*a*^*p* < 0.05, Bonferroni corrected. ^*b*^*p* < 0.05, uncorrected.*

## Discussion

Our study explored the potential mechanism of smoking effects on intrinsic brain activity in aMCI patients. The main results showed that non-smoking aMCI had the lowest ReHo in SMG and AG among groups, while smoking aMCI had greater ReHo in SMG and AG than non-smoking aMCI. Further, increased ReHo is related to better behavioral performance. Our results suggested that smoking might expose temporary compensatory response or protective effect on the intrinsic brain activity in aMCI patients.

First, aMCI patients (smoking and non-smoking) have lower left AG ReHo than controls. The AG is located in the IPL, an essential part of the default mode network (DMN) (Seghier, 2013). Based on the same method, previous aMCI studies also reported consistent results, indicating that ReHo mapping is repeatable (Cai et al., 2018). Then, we found that smoking aMCI had higher left SMG and AG ReHo than non-smoking aMCI and even higher left SMG ReHo than controls. Several studies have demonstrated that smoking might have protective effects on the neurodegenerative disease (Vanacore et al., 2000; Ascherio and Schwarzschild, 2016; Gallo et al., 2019). As the main ingredient of tobacco, nicotine is a cholinergic agonist lacking selectivity for most acetylcholine receptors (AChRs) (Valles et al., 2014). Most neuronal nicotinic AChRs (nAChRs) in the central nervous system (CNS) are excitatory. Since nicotine is not a substrate for acetylcholinesterase, it causes prolonged activation of nAChRs (Penton and Lester, 2009); nicotine can improve working memory function, learning, and attention, especially through α4β2 and α7 nAChRs (Levin et al., 2006). Animal research results showed that nicotine could reduce cognitive deficits (Koukouli et al., 2017). Many humans and animal models demonstrated that cognitive improvement is induced by nicotine (Levin et al., 2006). Long-term exposure to nicotine usually leads to an activity increase, or amount upregulation, of nAChR subtypes or subunits (Lombardo and Maskos, 2015; Liu and Li, 2018). Longitudinal studies are needed to assess the effects of smoking on brain function and the association of smoking with the risk of aMCI progressing to AD.

Some researchers found that increased brain activity is related to Aβ deposition. A spatially localized increase in the BOLD contrast reflects an increase in neural activity (Logothetis et al., 2001). There is a feedback loop between synaptic activity and Aβ. Neuronal hyperactivity may overcome the Aβ-induced block in learning and memory temporally. When neuronal activity levels increase too much, a physiological increase in Aβ can desensitize synapses and restore normal synaptic activity levels (Stargardt et al., 2015). RsfMRI studies suggested that the IPL ReHo increases in the aMCI patients but decrease in the AD patients (Zhang et al., 2012). In the early AD stage, increased regional brain activity may represent the compensatory effects for cognitive function impairment. Taken together, we speculated that increased ReHo might reflect the synergy effects between smoking and AD-related pathologies in smoking aMCI patients. Our correlation analysis result also supports our hypothesis. Specifically, the left SMG ReHo is positively related to memory, and the left AG ReHo is positively associated with visuospatial, memory, and general cognitive function.

Both SMG and AG are located in the inferior parietal lobes (IPL), an essential part of the default mode network (DMN). In detail, IPL is vulnerable to amyloid deposition at an early stage. Besides, smoking might also directly promote the amyloid pathway (Aβ oligomer production and extracellular fibrous Aβ aggregation) and abnormal tau phosphorylation through oxidative stress. Specifically, the p53 protein encoded by the p53 gene is pro-apoptotic and plays a crucial role in oxidative stress-dependent apoptosis. Previous studies confirmed that smoking amounts correlated with p53 mutations (Kondo et al., 1996). Other studies have pointed out that AD risk gene TREM2 is regulated by p53 tumor suppressor protein, and TREM2 is a target gene of p53 (Zajkowicz et al., 2018). Studies have found elevated p53 levels in the IPL of AD and aMCI patients (Cenini et al., 2008). On the other hand, the S-glutathionylation protein is a reversible binding process between glutathione and protein thiols (PSH) and protein redox regulation. The levels of p53 dimer and p53 monomer in the IPL of AD patients increased significantly, but the active form, p53 tetramer, was lacking. The glutathionylation of p53 may cause the negative regulation of the p53 tetramer. In contrast, p53 monomeric and dimeric may expose the reactive cysteine of the p53 nucleus to the external environment, making it more vulnerable to oxidative stress (Di Domenico et al., 2009). We speculate that smoking-related effects on cognitive function in aMCI patients may be related to oxidative stress caused by smoking, and the function of the IPL is susceptible. This conjecture needs to be confirmed by further basic research.

Notably, regions affected by the interaction effects between smoking and AD pathologies were all on the left hemisphere. Previous morphological studies showed that cerebral atrophy and neurodegenerative diseases in the early AD stage are preferentially affected in the left hemisphere (Janke et al., 2001). Some structural MRI studies showed that atrophy on the left hippocampus and entorhinal cortex *in APOE*ε*4* carriers (risk factors for AD) was quicker than the right (Jak et al., 2007; Donix et al., 2013). The result of an rs-fMRI study also points out that the differences between the *APOE*ε*4* carriers and the healthy population in the eigenvector centrality were all concentrated in the left hemisphere (Luo et al., 2017). Accordingly, we inferred that smoking exacerbated the AD pathologies accumulation on the left side to some extent.

## Limitations

Our study also had several limitations. First, due to the limited sample size of the ADNI database, the sample size of aMCI smokers is small. Future studies with larger sample size are needed to verify our work. Second, we only explored the effects of smoking on aMCI rather than healthy controls due to the small smoking sample in the control group. Thus, future studies are needed to include smokers with normal cognitive function to study the interaction between smoking and cognitive function. Moreover, future studies with larger sample size and available Fagerström Test for Nicotine Dependence (FTND) are needed to verify our work and the quantitative analysis should be used to explore the influence of smoking volume further and smoking time on the brain function of aMCI. The threshold *p* < 0.05 used in the statistical analysis of the article is not strict enough.

## Conclusion

Our study explored the possible mechanism by which smoking affects cognitive function from the perspective of ReHo. We believe that smoking has a more significant effect on the left IPL. In the aMCI patients, the left SMG and AG ReHo of the smoking population were higher than that of the non-smoking population. Paying attention to the changes in memory function and the visual–spatial function of smokers may help in the early detection of aMCI.

## Data Availability Statement

The data used in the preparation of this article were obtained from the Alzheimer’s disease Neuroimaging Initiative (ADNI) database: http://adni.loni.usc.edu/.

## Ethics Statement

Written informed consent was obtained from the individual(s) for the publication of any potentially identifiable images or data included in this article.

## Author Contributions

TZ contributed to statistic analysis and article writing. XL contributed to research technique and article modifying. QZ contributed to research technique. YF and ZYL contributed to literature searching. KL, XCL, and PH contributed to research technique supporting. YC contributed to article modifying. MZ and ZRL contributed to topic selection and article modifying. All authors contributed to the article and approved the submitted version.

## Conflict of Interest

The authors declare that the research was conducted in the absence of any commercial or financial relationships that could be construed as a potential conflict of interest.

## Footnotes

1 adni.loni.usc.edu
