# Supplementary material for: Effects of Smoking on Regional Homogeneity in Mild Cognitive Impairment: A Resting-State Functional MRI Study
Source: Front Aging Neurosci. 2020 Nov 19;12:572732. doi: 10.3389/fnagi.2020.572732 (PMC7717978; doi:10.3389/fnagi.2020.572732)
Supplement: Supplementary file 1 [file Data_Sheet_1.pdf]

Supplementary Material 1

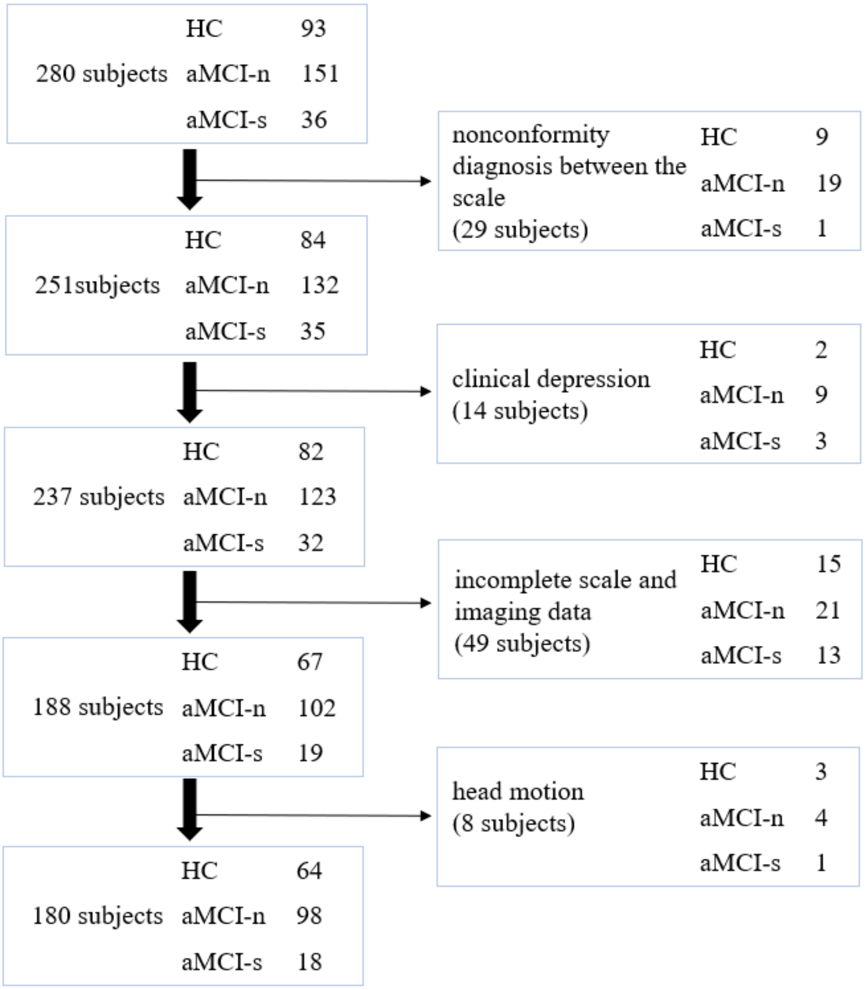

Fig.1 The screening processes of smoking subjects from the ADNI database

We excluded 29 subjects due to nonconformity diagnosis between the scale, 14 subjects due to clinical depression, 49 subjects due to incomplete scale and imaging data, 8 subjects due to head motion. Finally, 64 healthy controls, 18 smoking aMCI, and 98 non-smoking aMCI entered subsequent analyses.

Abbreviation: HC: healthy controls, aMCI: amnestic mild cognitive impairment

Table 1. The smoking information for some subjects

Subject	Description of smoking information
2022	Smoking 1 pack per day for 20 years
2121	Smoking from 1958 to 1960
2130	Average 0.5 packs per day for 3 years. Has not smoked for the last 46 years
2308	Smoking from 1951
2324	Smoking 1.5 packs per day for ten years, and quitted 15 years ago
2351	Smoked 1 pack per day for 40 years, and quitted in 1981
2379	Smoked half a pack per day for 8 years, and quitted 43 years ago
4005	Smoked about 3/4 - 1 packs per day for 15 years, and quitted 15 years ago
4029	Smoking for 8 years, and quitted in 1981
4294	Smoking half a pack per day
4332	Pipe and cigarette smoking
4417	Smoking 3 packs per day
4431	Cigarette and cigar smoking
4434	Smoking for 50 years
4476	Smoked 1 pack per day for 15 years and quitted 51 years ago
4674	Smoked 3 packs per day for 10 years and quitted in 1964
4891	Previous smoker
4947	Smoked 1/2 to 1 year

Supplementary Material 2

Table 2. The normal distribution of all neuropsychiatric scales

	Normal Parameters	*p*-value
**MMSE**	28.61 ± 1.55	*p* <0.001
**CDT**	4.6 ± 0.76	*p* <0.001
**IST**	12.86 ± 4.38	0.05
**DST**	11.25 ± 4.77	0.09
**AVLT**	41.06 ± 12.33	0.06
**SVF**	20.07 ± 5.01	0.01
**TMT-A**	34.43 ± 12.48	*p* <0.001
**TMT-B**	92.18 ± 55.41	*p* <0.001

Abbreviation: MMSE: Mini-Mental State Examination, CDT: clock drawing test, IST: immediate story retell, DST: delayed story retell, AVLT: auditory verbal learning test, SVF: semantic verbal fluency, TMT-A: Trail-Making Test, Part A, TMT-B: Trail-Making Test, Part B

Table 3. Test of Homogeneity of Variances

	Levene Statistic	DOF1	DOF2	*p*-value
**Age**	1.768	2	177	0.174
**Education**	0.861	2	177	0.424
**MMSE**	1.809	2	177	0.167
**CDT**	9.953	2	177	0.000
**IST**	2.673	2	177	0.072
**DST**	1.724	2	177	0.181
**AVLT**	0.072	2	177	0.930
**SVF**	0.191	2	177	0.826
**TMT-A**	0.634	2	177	0.531
**TMT-B**	0.515	2	177	0.599
**AG ReHo**	1.441	2	177	0.239
**SMG ReHo**	1.209	2	177	0.301

Abbreviation: MMSE: Mini-Mental State Examination, CDT: clock drawing test, IST: immediate story retell, DST: delayed story retell, AVLT: auditory verbal learning test, SVF: semantic verbal fluency, TMT-A: Trail-Making Test, Part A, TMT-B: Trail-Making Test, Part B

Supplementary Material 3

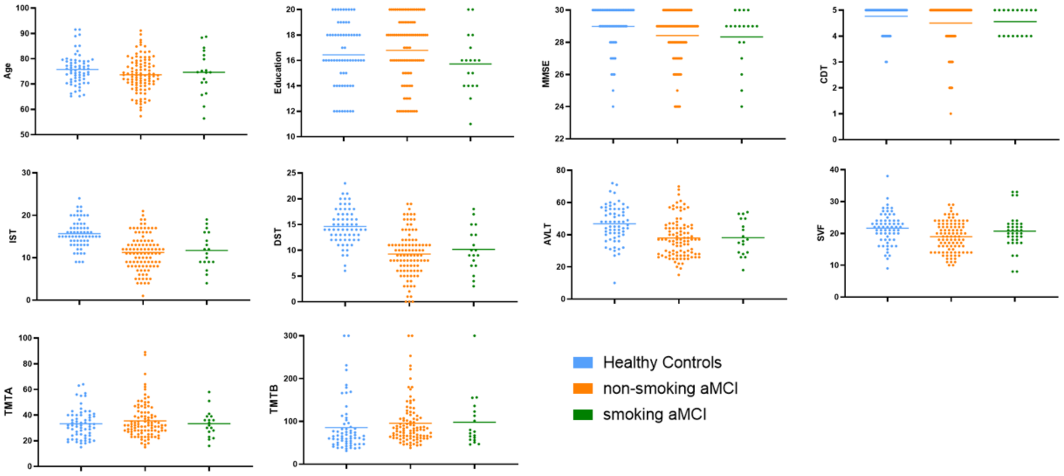

Fig.2 The scatter plot of each group on demographic information and neuropsychological scale

The figure shows the distribution of each group on demographic information and neuropsychological scale. Healthy controls had higher scores of the IST, DST, and AVLT than aMCI groups. However, no difference between smoking aMCI and non-smoking aMCI were found.

Abbreviation: MMSE: Mini-Mental State Examination, CDT: clock drawing test, IST: immediate story retell, DST: delayed story retell, AVLT: auditory verbal learning test, SVF: semantic verbal fluency, TMT-A: Trail-Making Test, Part A, TMT-B: Trail-Making Test, Part B, aMCI: amnestic mild cognitive impairment

Supplementary Material 4

Table 4. Brain areas with significant ReHo difference among the three groups

Region	Cluster Voxels	MNI Coordinate			Peak Intensity
		X	Y	Z	
**ANCOVA between three groups**					
**Left SMG**	241	-63	-21	33	9.88
**Right SMG**	54	63	-27	39	9.60
**Precentral Gyrus**	64	57	-6	12	8.99

MNI： Montreal Neurological Institute; X Y Z coordinates of the primary peak locations in the MNI space; SMG: supramarginal gyrus; AG: angular gyrus

Supplementary Material 5

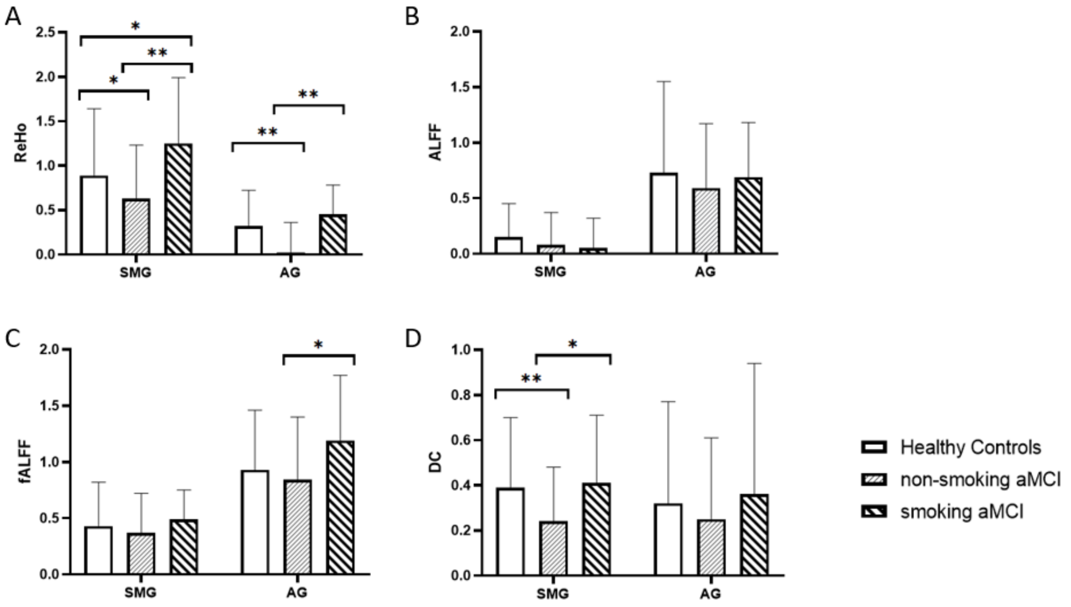

Fig.3 The trends of ReHo, ALFF, fALFF, and Degree Centrality in SMG and AG

Figures A, B, C, and D respectively represent the trends of ReHo, ALFF, fALFF, and DC in SMG and AG of the healthy controls, aMCI non-smoking group, and aMCI smoking group.

* $p < 0.05$, LSD corrected; ** $p < 0.01$, LSD corrected

Abbreviation: ReHo: regional homogeneity, ALFF: the amplitude of low-frequency fluctuation, fALFF: fractional ALFF, DC: Degree Centrality, SMG: supramarginal gyrus, AG: angular gyrus, aMCI: amnestic mild cognitive impairment

Supplementary Material 6

Table 5. Comparison of demographic information, behavioral data among three groups

	Non-smoking HC (n=64)	Smoking HC (n=8)	Non-smoking aMCI (n=98)	Smoking aMCI (n=18)	F/ χ2-value	*p*-value
**Age (years)**	75.75 ± 5.98	78.34 ± 5.70	73.67 ± 6.78	74.61 ± 8.84	2.09	0.10
**Education (years)**	16.44 ± 2.50	16.63 ± 2.33	16.80 ± 2.60	15.72 ± 2.32	0.99	0.40
**Sex (F/M)**	37/27	1/7	39/59	4/14	12.34	0.006[*]
**MMSE**	28.98 ± 1.37	28.88 ± 0.64	28.42 ± 1.59	28.33 ± 1.75	2.13	0.10
**Visuospatial**						
**CDT**	4.77 ± 0.50	4.75 ± 0.46	4.50 ± 0.91	4.56 ± 0.51	1.77	0.15
**Memory**						
**IST**	15.67 ± 3.28	15.50 ± 3.82	11.24 ± 4.15	11.72 ± 4.28	18.68	< 0.001[ab]
**DST**	14.63 ± 3.45	14.38 ± 4.24	9.25 ± 4.38	10.16 ± 4.31	24.47	< 0.001[ab]
**AVLT**	46.72 ± 11.51	39.00 ± 9.32	37.92 ± 11.86	38.06 ± 10.98	7.99	< 0.001[ab]
**Language**						
**SVF**	21.63 ± 4.91	19.50 ± 3.42	18.95 ± 4.68	20.67 ± 5.77	4.10	0.008[a]
**Attention**						
**TMT-A**	33.22 ± 11.45	32.88 ± 5.96	35.44 ± 13.45	33.28 ± 10.42	0.52	0.67
**Execution**						
**TMT-B**	85.41 ± 59.33	75.00 ± 25.00	95.54 ± 51.54	97.94 ± 62.08	0.77	0.51

Data are presented as mean ± standard deviation, HC: healthy controls, aMCI: amnestic mild cognitive impairment, F: female, M: male, MMSE: Mini-Mental State Examination, CDT: clock drawing test, IST: immediate story retell, DST: delayed story retell, AVLT: auditory verbal learning test, SVF: semantic verbal fluency, TMT-A: Trail-Making Test, Part A, TMT-B: Trail-Making Test, Part B

[*] Because of the small number of female smokers, we will further use ANCOVA to eliminate the effect of sex.

[a] Post hoc paired comparisons showed significant group differences between non-smoking HC and non-smoking aMCI, after LSD correction. [b] Post hoc paired comparisons showed significant group differences between HC and aMCI, after LSD correction.

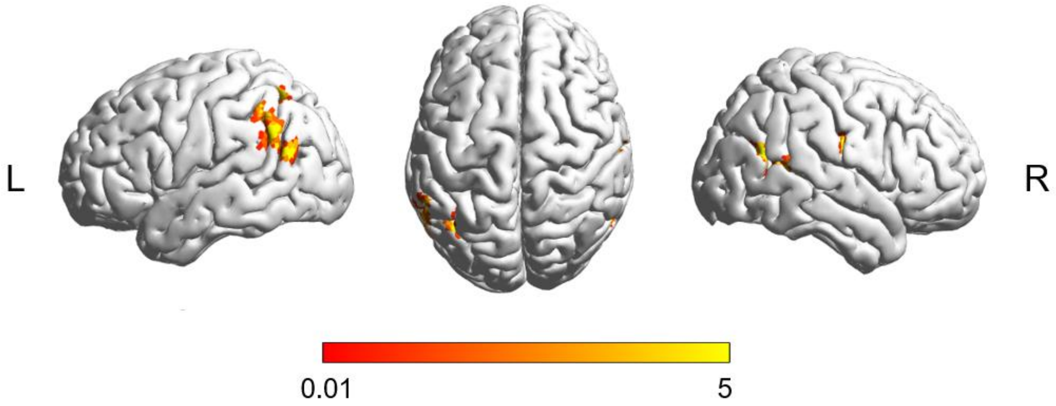

Fig.4 The ReHo difference among the four groups

The figure shows the difference in the ReHo index after adjustment for age, sex, education, and gray matter volume in healthy non-smoking controls, smoking healthy controls, non-smoking aMCI, and smoking aMCI group. The four groups have significant differences in the left SMG, the left AG, the right precentral gyrus, and the right superior temporal (GRF corrected, $p < 0.05$ at height and $p < 0.05$ at cluster level).

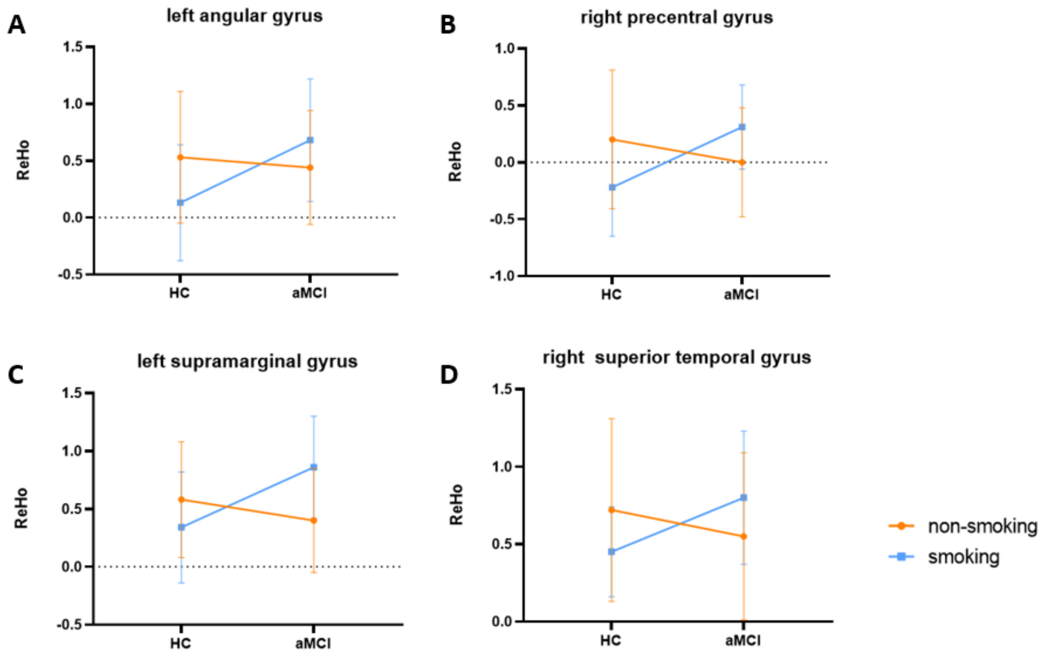

Fig.5 The interactions on ReHo between cognitive and smoking

The figure shows the ReHo of aMCI people was lower than NC among non-smokers, while the ReHo of MCI was higher among smokers. In cognitively healthy populations, the ReHo of the smokers was lower. In aMCI populations, compared with the non-smokers, the ReHo of the smokers increased instead.

Abbreviation: Abbreviation: HC: healthy controls, aMCI: amnestic mild cognitive impairment, ReHo: regional homogeneity

Supplementary Material 7

Table 6. Correlations between ReHo and neuropsychological scores in each group

	Left SMG						Left AG					
	**NC**		non-smoking aMCI		smoking aMCI		**NC**		non-smoking aMCI		smoking aMCI	
	correlation coefficient	*p*-value	correlation coefficient	*p*-value	correlation coefficient	*p*-value	correlation coefficient	*p*-value	correlation coefficient	*p*-value	correlation coefficient	*p*-value
**MMSE**	-0.13	0.31	0.08	0.44	-0.25	0.31	0.02	0.85	0.30	0.002	-0.16	0.52
**Visuo-spatial**												
**CDT**	-0.13	0.13	-0.00	0.99	-0.23	0.36	0.15	0.23	0.16	0.11	0.36	0.14
**Memory**												
**IST**	0.16	0.21	0.13	0.20	-0.07	0.80	0.44	<0.001	0.20	0.05	0.10	0.69
**DST**	0.16	0.21	0.11	0.28	-0.01	0.96	0.31	0.01	0.17	0.10	0.22	0.38
**AVLT**	0.09	0.50	0.16	0.11	-0.12	0.63	0.00	0.98	0.23	0.02	-0.04	0.89
**Language**												
**SVF**	-0.08	0.56	0.09	0.36	-0.04	0.87	0.00	0.98	0.11	0.28	-0.19	0.45
**Attention**												
**TMT-A**	-0.03	0.79	0.06	0.55	0.15	0.57	-0.04	0.74	-0.04	0.68	-0.08	0.74
**Execution**												
**TMT-B**	0.16	0.21	-0.02	0.87	0.41	0.09	-0.15	0.23	-0.077	0.45	0.21	0.41

MMSE: Mini-Mental State Examination, CDT: clock drawing test, IST: immediate story retell, DST: delayed story retell, AVLT: auditory verbal learning test, SVF: semantic verbal fluency, TMT-A: Trail-Making Test, Part A, TMT-B: Trail-Making Test, Part B

The left AG ReHo is related to immediate recall, delayed recall in healthy controls ($p < 0.01$) and is related to MMSE and AVLT in the non-smoking aMCI ($p < 0.05$).

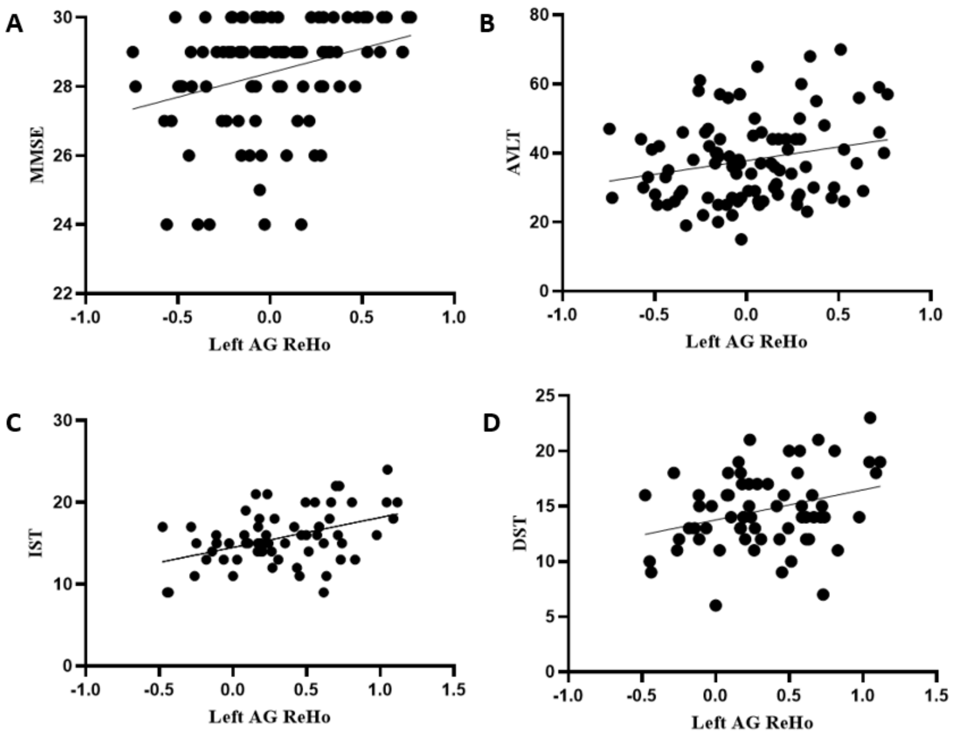

Fig.6 Correlation between ReHo and neuropsychological scores in healthy controls and non-smoking aMCI

As shown in the figure, Figures A and B represent the correlation between the left AG ReHo and MMSE, AVLT in healthy controls. And Figures C and D shows the correlation between the left AG ReHo and immediate recall and delayed recall. All of neuropsychological scores are positively correlated ($p < 0.05$). The abscissa indicates the left AG ReHo, and the ordinate indicates the corresponding neuropsychological scale score.

Supplementary Material 8

Table 7. Correlations among the years and the total amount of smoking and ReHo and neuropsychological scores

	the years of smoking		the total amount of smoking	
	**correlation coefficient**	***p*-value**	**correlation coefficient**	***p*-value**
**MMSE**	0.454	0.138	0.357	0.385
**CDT**	0.482	0.113	0.431	0.286
**IST**	-0.259	0.417	-0.103	0.809
**DST**	-0.085	0.793	-0.138	0.744
**AVLT**	-0.083	0.797	-0.15	0.723
**SVF**	0.398	0.200	0.492	0.215
**TMT-A**	-0.259	0.417	-0.301	0.469
**TMT-B**	-0.234	0.464	0.058	0.892
**left SMG ReHo**	-0.404	0.193	-0.495	0.212
**left AG ReHo**	0.053	0.869	-0.194	0.645

Abbreviation: MMSE: Mini-Mental State Examination, CDT: clock drawing test, IST: immediate story retell, DST: delayed story retell, AVLT: auditory verbal learning test, SVF: semantic verbal fluency, TMT-A: Trail-Making Test, Part A, TMT-B: Trail-Making Test, Part B, SMG: supramarginal gyrus, AG: angular gyrus, ReHo: regional homogeneity
